# Functional loss of ERBB receptor feedback inhibitor 1 (MIG6) promotes glioblastoma tumorigenesis by aberrant activation of epidermal growth factor receptor (EGFR)

**DOI:** 10.1002/1878-0261.13717

**Published:** 2024-08-11

**Authors:** Sang Ah Yi, Daseul Cho, Sujin Kim, Hyunjin Kim, Myung Kyung Choi, Hee Seong Choi, Sukjin Shin, Sujin Yun, Ahjin Lim, Jae Kyun Jeong, Da Eun Yoon, Hye Ji Cha, Kyoungmi Kim, Jeung‐Whan Han, Hyun‐Soo Cho, Jeonghee Cho

**Affiliations:** ^1^ Epigenome Dynamics Control Research Center, School of Pharmacy Sungkyunkwan University Suwon Korea; ^2^ Department of Biomedical Science & Engineering Dankook University Cheonan Korea; ^3^ Department of Nanobiomedical Science Dankook University Cheonan Korea; ^4^ Department of Systems Biology, College of Life Science and Biotechnology Yonsei University Seoul Korea; ^5^ Department of Biomedical Sciences Korea University College of Medicine Seoul Korea; ^6^ Department of Physiology Korea University College of Medicine Seoul Korea; ^7^ Present address: Chemical Biology Program Memorial Sloan Kettering Cancer Center New York NY USA

**Keywords:** biomarker, EGFR, glioblastoma (GBM), MIG6, targeted cancer therapy

## Abstract

Dysregulation of epidermal growth factor receptor (EGFR) is one of the most common mechanisms associated with the pathogenesis of various cancers. Mitogen‐inducible gene 6 [MIG6; also known as ERBB receptor feedback inhibitor 1 (ERRFI1)], identified as a feedback inhibitor of EGFR, negatively regulates EGFR by directly inhibiting its kinase activity and facilitating its internalization, subsequently leading to degradation. Despite its proposed role as an EGFR‐dependent tumor suppressor, the functional consequences and clinical relevance in cancer etiology remain incompletely understood. Here, we identify that the stoichiometric balance between MIG6 and EGFR is crucial in promoting EGFR‐dependent oncogenic growth in various experimental model systems. In addition, a subset of *ERRFI1* (the official gene symbol of MIG6) mutations exhibit impaired ability to suppress the enzymatic activation of EGFR at multiple levels. In summary, our data suggest that decreased or loss of MIG6 activity can lead to abnormal activation of EGFR, potentially contributing to cellular transformation. We propose that the mutation status of *ERRFI1* and the expression levels of MIG6 can serve as additional biomarkers for guiding EGFR‐targeted cancer therapies, including glioblastoma.

## Graphical abstract

EGFR dysregulation is one of the most common mechanisms in glioblastoma pathogenesis. Here, we identified that reduced MIG6 activity, stemming from lower protein expression or specific *ERRFI1* loss‐of‐function mutations, causes aberrant EGFR activation by impairing multiple negative‐feedback‐inhibition mechanisms. This finding suggests that *ERRFI1* mutation status and MIG6 expression levels could potentially serve as biomarkers for guiding EGFR‐targeted glioblastoma therapies.

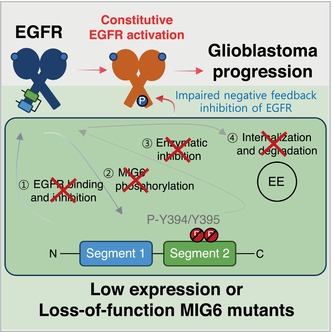

**Abbreviations**
ATP: adenosine triphosphate
BRAF: B‐Raf proto‐oncogene
cDNA: complementary DNA
COSMIC: catalogue of somatic mutations in cancer
EGF: epidermal growth factor
EGFR: epidermal growth factor receptor
ERBB2: Erb‐B2 receptor tyrosine kinase 2
ERKs: extracellular signal‐regulated kinases
*Errfi1*
: ERBB receptor feedback inhibitor 1
GBM: glioblastoma
GLICO: cerebral organoid glioma
GST: glutathione S‐transferase
KRAS: Kirsten rat sarcoma virus
Mig6: mitogen‐inducible gene 6
neoCOR: neoplastic cerebral organoid
PIK3CA: phosphatidylinositol‐4,5‐bisphosphate 3‐kinase catalytic subunit alpha
shRNA: short hairpin RNA
STAT5: signal transducer and activator of transcription 5
TCGA: The Cancer Genome Atlas

## 1 Introduction

The aberrant activation of epidermal growth factor receptor (EGFR), a member of the ErbB family, and the consequent abnormal activation of its downstream signaling pathways are widely recognized as hallmarks in the pathogenesis of various cancers, including lung adenocarcinoma, glioblastoma (GBM), and colorectal cancers [1, 2, 3, 4]. The oncogenic activation of EGFR results from diverse molecular mechanisms, including various genomic alterations such as somatic mutations and local amplification/deletion within the *EGFR* gene, and overexpression of EGFR [5, 6, 7, 8, 9]. Importantly, a remarkable correlation has been observed between the specific oncogenic EGFR mutants resulting from distinct activation mechanisms and the effectiveness of EGFR‐targeted therapies, including tyrosine kinase inhibitors (TKIs) and EGFR‐targeted monoclonal antibodies (mAbs) [8, 10, 11, 12, 13, 14, 15]. Therefore, a more comprehensive and thorough understanding of the various molecular mechanisms leading to the oncogenic activation of EGFR is essential for improving the efficacy of current EGFR‐targeted therapy in clinical settings.

Mitogen‐inducible gene 6 (MIG6) is well established as a crucial negative regulator of EGFR and other members of the ErbB family through several coordinated mechanisms [16, 17, 18, 19, 20, 21, 22]. The proposed functions of MIG6 include (a) direct binding to EGFR, inhibiting the formation of asymmetric dimers of EGFR receptors via the MIG6 segment 1 domain, (b) direct suppression of substrate access to EGFR by phosphorylated MIG6 at Y394 and Y395 residues on the MIG6 segment 2 domain, and (c) promoting internalization and degradation of EGFR [17, 18, 19, 21, 22]. Considering the critical role of MIG6 segment 1/2 domains and Y394/Y395 phosphorylation on segment 2 in negatively regulating EGFR, it is hypothesized that dysfunction of MIG6 could lead to a failure in the feedback inhibitory role against activated EGFR [19]. This could contribute to the oncogenic activation of EGFR signaling, even in the absence of genomic alterations affecting EGFR. In fact, the loss of *Errfi1* (ERBB receptor feedback inhibitor 1, official gene name of MIG6) in mice leads to a significant increase in neoplastic lesions due to aberrant activation of EGFR signaling [20, 23, 24]. In addition, numerous evidence has shown that the functional loss of MIG6 is strongly associated with tumor cell growth, proliferation, invasiveness, and resistance to hormonal therapy in various cancers [25, 26, 27, 28]. Moreover, the genomic analysis of human gliomas revealed frequent focal deletions of *ERRFI1*, suggesting that the loss of MIG6 activity may contribute to the development of GBM through aberrant activation of EGFR [19, 21].

In this study, we demonstrate the significant impact of the stoichiometric balance of MIG6‐EGFR on oncogenic potential. Furthermore, we identify a subset of loss‐of‐function *ERRFI1* mutations and characterize them to confirm that indeed the functional loss of MIG6 contributes to tumorigenesis in a manner dependent on dysregulated EGFR. These findings suggest that the *ERRFI1* mutation status, along with the levels of MIG6 proteins, could be a promising additional biomarker for selecting patients for EGFR‐targeted therapy.

## 2 Materials and methods

### 2.1 Genomic data analysis

*ERRFI1* mutations were assessed in pan cancer genomic databases, specifically in COSMIC (release v97, 29th November 2022 version) [29] and TCGAbiolinks (version 36) [30]. The data was filtered to include only missense or nonsense mutations that were annotated in proteins.

### 2.2 Cell culture

All cell lines used in this study were cultured in DMEM or RPMI‐1640 (Corning, New York, NY, USA), supplemented with FBS (cat. No. TMS‐013‐BKR, Merck, Darmstadt, Germany), BS (cat. No. 16170078, Gibco, Jenks, OK, USA), penicillin, streptomycin, and l‐glutamine (Gibco). A172 (RRID:CVCL_0131), Hs683 (RRID:CVCL_0844) and NIH‐3T3 (RRID:CVCL_0594) cells were purchased from the Korean Cell Line Bank (KCLB, Seoul, South Korea). HEK‐293T (RRID:CVCL_0063) and H226 (RRID:CVCL_1544) cells were purchased from the American Type Culture Collection (ATCC, Manassas, VA, USA). LN443 (RRID:CVCL_3960) and U251 (RRID:CVCL_0021) cells were kindly provided by Dr. Matthew Meyerson. LN18 (RRID:CVCL_0392), LN229 (RRID:CVCL_0393), U138 (RRID:CVCL_0020), U373 (RRID:CVCL_2219) and U87 (RRID:CVCL_0022) cells were kindly provided by Dr Do‐Hyun Nam. All experiments were performed with mycoplasma‐free cells. Short tandem repeat (STR) profiling was utilized to authenticate all the cell lines.

### 2.3 Generation of constructs

The N‐terminal Myc‐tagged WT MIG6 expression plasmid was generated in the pBabe‐puro vector through PCR‐based subcloning of MIG6 cDNA. The V5‐tagged EGFR and ERBB2 expression plasmids were generated in the PLX302 vector through gateway cloning with pDONOR EGFR and pDONOR ERBB2, respectively (Addgene, Watertown, MA, USA). The EGFRvIII and EGFR CTED8 expression plasmids were previously reported [31, 32]. The MIG6 and ERBB2 mutant‐expressing plasmids were created using the QuickChange site‐directed mutagenesis kit, with pBabe WT MIG6 and pLX302 ERBB2 as the respective templates following the manufacturer's protocol (Agilent Technologies, Santa Clara, CA, USA). A subset of MIG6 mutant‐expressing plasmids was subcloned into the PLX302 vector as well. Sanger sequencing was performed on the generated plasmids to validate the correct introduction of mutations. The GST‐MIG6 segment 1/2 (330–399) expression plasmid was generated by subcloning with pBabe MIG6 plasmid as a template into the pGEX4T‐1 vector pre‐cleaved with restriction enzymes XhoI (NEB, Ipswich, MA, USA) and BamHI (NEB).

### 2.4 Expression constructs

The expression plasmids for wild‐type (WT) EGFR, EGFR L858R mutant, WT MIG6, and MIG6 Y394F/Y395F mutant were previously described [19].

### 2.5 Generation of cell lines by viral transduction

All cell lines ectopically expressing EGFR and/or MIG6 were generated by viral infection of pBabe‐puro or PLX302 plasmids containing the respective genes, and the infected cells were subsequently pooled together as previously described [6, 33]. The methods to produce virus, infection, and maintenance of the established cell lines have been previously described [34]. For RNAi, pLKO.shRNA were purchased from Sigma (St. Louis, CA, USA) and produced using protocols from the RNAi Consortium (https://portals.broadinstitute.org/gpp/public/). The shRNA constructs used were EGFR #a (TRCN0000010329), EGFR #b (TRCN0000121068), MIG6 #a (TRCN0000118128), and MIG6 #b (TRCN0000291948). For infection, cells were incubated for 8 h with diluted virus‐containing media supplemented with 8 μg·mL^−1^ polybrene (Sigma). Subsequently, the infected cells were subjected to puromycin treatment for 1 week.

### 2.6 Transient transfection

HEK‐293T cells were seeded at a density of 3 × 10^5^ cells per well in 6‐well plates. After 24 h, we performed transient transfections of the plasmids using FuGENE Transfection Reagent (Promega, Madison, WI, USA), following the manufacturer's instructions. After 48 h, the transfected cells were serum‐starved overnight and then either treated with EGF (10 ng·mL^−1^) or left untreated. Subsequently, the cells were lysed in RIPA buffer for further analysis.

### 2.7 Immunoblotting, immunoprecipitation, and antibodies

Cells were lysed using either RIPA buffer or NP40 buffer supplemented with sodium orthovanadate (0.2 mm), BGP (0.2 mm), aprotinin (0.5 μg·mL^−1^), Leupeptin (0.5 μg·mL^−1^), and PMSF (1 mm). For immunoprecipitation, 500–1000 μg of proteins were incubated with the specified antibodies and protein A agarose (Thermo Fisher Scientific, Waltham, MA, USA) for 14 h at 4 °C. Immunoprecipitation complexes were eluted by boiling in 2× SDS loading buffer and then subjected to immunoblotting analysis. The p‐MIG6 antibody used in this study was previously described [19]. Antibodies against p‐ERK1/2 (T202/Y204), p‐EGFR (Y1068), p‐STAT5 (Y694), ERBB2, p‐ERBB2 (Y1221), and vinculin were purchased from Cell Signaling Technology (Danvers, MA, USA). Anti‐EGFR and anti‐Myc tag antibodies were obtained from Bethyl laboratories (Montgomery, AL, USA). Anti‐β‐actin and anti‐V5 tag antibodies were sourced from Sigma. Buffer compositions are displayed in Table S1.

### 2.8 Anchorage‐independent growth assay

Soft agar assays were performed in the presence or absence of EGF (Gibco) as previously described [6]. After 2–3 weeks, the colonies formed in soft agar were quantified by analyzing photographed images of the soft agar using image j software (NIH, Bethesda, MD, USA). The data were presented as a relative ratio in a graph, normalized to the number of colonies formed by control cells. Each assay was conducted in triplicate and repeated a minimum twice.

### 2.9 Cell proliferation assay

The cell proliferation ability of GBM cell lines was evaluated by seeding 3 × 10^3^~2 × 10^4^ cells per well in 6‐well plates. After 24 h, the cells were treated with EGF (10 ng·mL^−1^). The cell number was counted every 2 or 3 days, and the reported results represent the average of three independent cell counts per well.

### 2.10 Cell viability assay

After 24 h of cell seeding in 96‐well plates, the cells were treated with afatinib (Selleckem, Houston, TX, USA) at the indicated concentrations and further incubated for 7 days. The viable cells were assessed using Cell Counting Kit‐8 solution (Dojindo, Rockville, MD, USA), and the absorbance was measured at 450 nm after 3 h. The data were expressed as percent growth relative to untreated control cells.

### 2.11 Expression and purification of EGFR kinase and MIG6 peptides

The human EGFR kinase domain (residue 696–1022) was cloned into the pFastBac1 vector as a fusion with N‐terminal 10xHis tag and Tobacco etch virus (TEV) protease cleavage site. EGFR kinase domain were expressed in *Spodoptera frugiperda* (Sf9) cells (2 × 10^6^ cells·mL^−1^, ESF921 medium) and lysed by sonication in lysis buffer. The supernatant fraction was isolated by centrifugation at 20 000 *g* at 4 °C for 1 h and filtered by 0.45 μm bottle top filter. The protein was incubated at 4 °C for 2 h with nickel‐affinity resin and eluted with elution buffer. After overnight cleavage with TEV protease, samples were further purified by size exclusion chromatography on Superdex 200 increase (Cytiva, Marlborough, MA, USA) with SEC buffer.

PCR fragments of wild‐type (WT) and mutant MIG6 regions, spanning residues 330–399 of human MIG6, were inserted into the pET28a vector. These cloned plasmids were expressed in *Escherichia coli* that were induced with 0.1 mm IPTG and cultured at 17 °C overnight. Purification method was same with EGFR kinase. Each protein was concentrated and stored in aliquots at −80 °C until they were used. Buffer compositions are displayed in Table S1.

### 2.12 *In vitro* kinase assay

EGFR kinase activities were measured using the Kinase‐Glo® Luminescent Kinase assay kit (Promega) according to the manufacturer's instructions. The kinase reactions were performed using kinase reaction buffer in 96‐well white plates (cat. No.20196, SPL, Pocheoon, South Korea). Unincubated assays were performed in 30 μL volumes of 7.8 nm EGFR kinase domain protein, 50 μm ATP, and 20 μL of 1 μm MIG6 peptide variants.

Pre‐incubated assays were performed in 30 μL volumes of 7.8 nm EGFR kinase domain protein, 50 μm ATP, and 1 μm MIG6 WT peptide. The pre‐kinase reaction was incubated at room temperature for 30 min and additional reaction 30‐min reaction time was given again after supplement 1 μm MIG6 peptide variants. The luminescent signal was read after adding 50 μL of Kinase‐Glo reagent and incubating at room temperature for 10 min. The luminescence was measured using a luminometer (CLARIOstar Plus multilabel plate reader, BMG LABTECH) and normalized with the luminescence signal when mixing ATP with MIG6 peptide in the absence of EGFR kinase domain protein. All kinetic data were calculated using graphpad prism software 7.0 (GraphPad Software, Boston, MA, USA).

### 2.13 Immunocytochemistry

Immunocytochemistry was performed with fixed A172 cells as previously described [35]. The cells were fixed with 4% paraformaldehyde for 15 min, washed with PBS, and permeabilized with 0.1% Triton X‐100 for 15 min. After the blocking with 0.1% bovine serum albumin (BSA) at room temperature for 1 h, the cells were incubated with primary antibodies diluted in 0.1% BSA overnight at 4 °C, followed by incubation with secondary antibodies diluted (1 : 600) at room temperature for 1 h. After three washes with PBS, the cells were incubated with DAPI (1 : 1000) at room temperature for 15 min. The fluorescence images were taken by the Cytation™ 5 Cell Imaging Multi‐Mode Reader (BioTek, Winooski, VT, USA) and quantified with image j.

### 2.14 Generation of human brain organoids

H7 human embryonic stem cells (hESCs) were differentiated into dorsal forebrain organoid as previously described [35]. On day 0, H7 cells were detached from plate and reaggregated in ultra‐low‐cell adhesion 96 well V‐bottom plates (1 × 10^4^ cells per well) with cortical differentiation medium (CDM) I (Glasgow‐MEM containing 20% KSR, 0.1 mm MEM‐NEAA, 1 mm sodium pyruvate, 0.1 mm β‐mercaptoethanol, and P/S). 20 μm Y‐27632 was added to CDM I for day 0–6.3 μm IWR‐1 and 5 μm SB431542 were added to CDM I for day 0–18. From day 18 to day 34, the aggregates were moved to 60 mm ultra‐low‐attachment plates on the orbital shaker in CDM II (DMEM/F12 containing 2 mm glutamax, 1% N_2_, 1% CD lipid concentrate, 0.25 μg·mL^−1^ fungizone, and P/S). From day 35 to day 69, the organoids were incubated in CDM III (CDM II supplemented with 10% FBS, 5 μg·mL^−1^ heparin, and 1% Matrigel). From day 70, the organoids were incubated in CDM IV (CDM III supplemented with B27 and 2% Matrigel). CDM I was changed every 3 days while CDM II, CDM III, and CDM IV were changed every 2–3 days.

### 2.15 Generation of glioblastoma‐brain organoid model

To generate GBM cells that stably express eGFP, A172 and U251 cells were infected with eGFP‐expressing AAV2 viral particles (catalog No. AA001, Gene Copoeia, Rockville, MD, USA), and sorted using fluorescence‐activated cell sorting. To establish the GBM‐brain organoid model, A172 or U251 cells were initially aggregated in ultra‐low‐cell adhesion 96‐well V‐bottom plates at a density of 1 × 10^3^ to 2 × 10^3^ cells per well for 2 days. These tumor spheroids were co‐cultured with brain organoids in 12‐well ultra‐low‐attachment plates on an orbital shaker. Images of GBM spheroids attached and integrated into brain organoids were captured using the Cytation™ 5 Cell Imaging Multi‐Mode Reader (BioTek).

### 2.16 Statistical analysis

Statistical significance was assessed using the two‐tailed Student's *t*‐test, and the results were evaluated based on *P*‐values. Results with * (*P* < 0.05), ** (*P* < 0.01), and *** (*P* < 0.001) were considered statistically significant.

### 2.17 Ethics

No human subject or animal was used in this study.

## 3 Results

### 3.1 The expression levels of MIG6 correlate with cell proliferation and oncogenic potentials of glioblastoma cell lines in an EGFR‐dependent manner

**Fig. 1:**
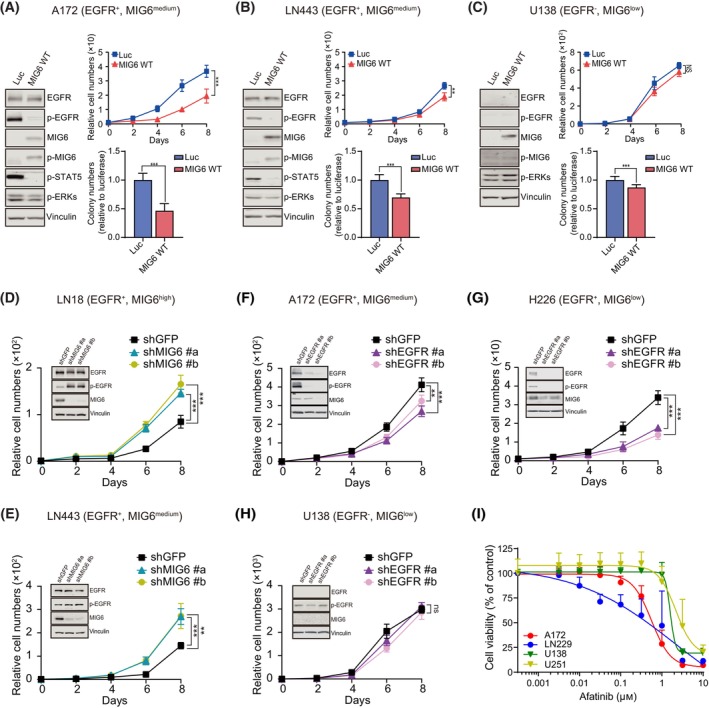
The stoichiometric ratio of MIG6 and EGFR expression correlates with EGFR‐dependent oncogenic growth in a subset of glioblastoma and lung cancer cell lines. (A–C) Graphs show relative cell numbers of A172 (A), LN443 (B), and U138 (C) cells expressing wild‐type (WT) MIG6 (red) or luciferase (Luc) (blue) over time, normalized by the number of cells at day 0, represented as the mean ± SD of octuplicate of two independent experiments (top right panel). The relative colony numbers formed in soft agar by the cells expressing WT MIG6 (red), quantified following 2 weeks and normalized with the colony numbers of the cells expressing Luc (blue) were depicted as the mean ± SD of triplicates in three independent experiments (bottom right panel). Immunoblotting images were obtained through the analysis of cell lysates prepared from the aforementioned cells, utilizing the indicated antibodies. The phosphorylated residues corresponding to the indicated proteins are detected using the following phospho‐specific antibodies: p‐EGFR (Y1092), p‐MIG6 (Y394/Y395), p‐STAT5 (Y694), and p‐ERKs (T202/Y204). (D, E) Relative cell numbers of LN18 (D) or LN443 cells (E) expressing either shRNAs targeting MIG6 (blue and green) or GFP (black) are shown at the indicated days, normalized to the cell numbers at day 0. Western blotting images obtained from lysates of each cells display the levels of MIG6 and p‐EGFR in those cells. (F–H) Following the same procedure as described in (D, E), the relative cell growth of A172 (F), H226 (G), or U138 (H) cells introduced with either shRNAs targeting EGFR (purple and pink) or GFP (black) was depicted, and the levels of EGFR in these cells were shown by western blotting analysis. (I) A172, LN229, U138, and U251 cells were treated with afatinib at the indicated concentrations for 7–8 days in the presence of EGF (10 ng·mL^−1^), and their cell viability was assessed. The results are presented in a graph as the mean ± SD of octuplicate wells in three independent experiments and Student's *t*‐test was used to determine statistical significance. **, *P* < 0.01; and ***, *P* < 0.001.

Given that the interaction of MIG6 with EGFR is crucial for its role in negatively regulating EGFR [19, 22], we investigated the functional significance of maintaining a stoichiometric balance between MIG6 and EGFR in relation to the oncogenic potential of GBM cell lines. We categorized nine glioblastoma cell lines into four groups based on the expression levels of EGFR and MIG6: (a) EGFR‐positive (EGFR^+^) and MIG6‐high level (MIG6^high^); (b) EGFR^+^ and MIG6‐medium level (MIG6^medium^); (c) EGFR^+^ and MIG6‐low level (MIG6^low^); (d) EGFR‐negative (EGFR^−^) and MIG6^low^ (Fig. S1A,B). Next, we modulated MIG6 expression in the selected cell lines (Fig. 1). Ectopic MIG6 expression in a subset of group 2 and 3 cell lines led to reduced cell proliferation and anchorage‐independent colony formation in soft agar, one of the hallmarks of cellular transformation (Fig. 1A,B and Fig. S1C,D). However, no such effects were found in U138 and U251 cells belonging to group 4 (Fig. 1C and Fig. S1E). The lowered oncogenic potential observed in ectopic MIG6 expressing cell lines is likely due to MIG6‐mediated diminished EGFR activity, as supported by increased levels of MIG6 and p‐MIG6, and decreased autophosphorylation of EGFR, and its downstream effectors, p‐ERKs or p‐STAT5 (Fig. 1A,B and Fig. S1C,D). Furthermore, similar anti‐oncogenic effects were observed in H226 lung cancer cells expressing MIG6 (Fig. S1F), where the *ERRFI1* gene is mutated, and MIG6 expression is absent [36]. Conversely, reducing MIG6 expression in LN18 and LN443 cell lines, which belong in group 1 and 2, respectively, led to increased cell proliferation along with enhanced EGFR signaling (Fig. 1D,E). Moreover, silencing EGFR in A172 and H226 cells, where MIG6 expression is relatively low and presumably EGFR activity is high, decreased cell proliferation (Fig. 1F,G). This result reflects the effect of MIG6 overexpression in the same cells as shown in Fig. 1A and Fig. S1F, indicating that indeed EGFR plays a crucial role in oncogenic growth in these cells (Fig. 1F,G). However, this effect was less apparent in U138 and U251 cells lacking EGFR and MIG6 expression (Fig. 1H and Fig. S1G).

Notably, A172 and LN229 cells, confirmed with EGFR‐dependent cell growth, exhibited higher sensitivity to afatinib, an EGFR and ERBB2 dual targeted drug, than U138 and U251 cells regardless of EGF treatment (Fig. 1I and Fig. S2A). The finding of increased afatinib sensitivity in ectopic MIG6‐expressing cells (Fig. S2B,C) supports the significance of EGFR as an oncogenic driver in A172 and LN229 cells and further highlights the potential clinical relevance of pharmacological EGFR suppression in the context of MIG6 expression.

Taken together, our data suggest that an imbalanced MIG6‐EGFR stoichiometric ratio can drive abnormal EGFR activation, contributing to oncogenic cell growth. This highlights EGFR's crucial role in oncogenesis, particularly in circumstances marked by low MIG6 expression. Thus, targeting EGFR in these conditions may hold promise as a novel therapeutic strategy.

### 3.2 A subset of patient‐derived *ERFFI1* mutations lose their ability to suppress EGFR activity

**Fig. 2:**
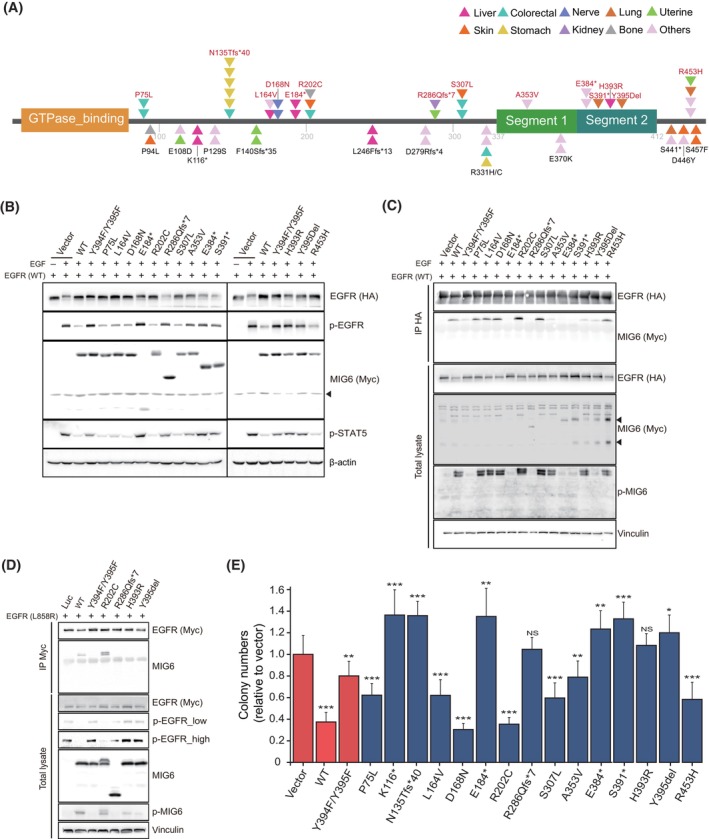
Functional characterization of *ERRFI1* mutations for their impact on EGFR suppression. (A) Schematic diagram showing identified *ERRFI1* mutations in the context of the *ERRFI1* domain structure, with triangles indicating the numbers of mutation cases found in various types of cancer, color‐coded accordingly. Thirteen mutations depicted on the upper side of the schematic were chosen for functional analysis. Asterisks indicate translational termination (stop) codon. (B) Cell lysates prepared from 293T cells co‐expressing HA‐tagged wild‐type (WT) EGFR and either Myc‐tagged WT MIG6 or selected MIG6 mutants, as indicated, following EGF treatment, were blotted with antibodies against HA, Myc, p‐EGFR, or p‐STAT5. β‐actin was used as a loading control. The experiment was repeated in triplicates. (C) Immunoprecipitation was conducted with an anti‐HA antibody using cell lysates from 293T cells as described in (B), followed by immunoblotting with the indicated antibodies. Vinculin was used as a loading control. The experiment was repeated in triplicates. (D) Cell lysates from 293T cells co‐expressing Myc‐tagged EGFR L858R mutant and either WT MIG6 or the indicated MIG6 mutants were used for immunoprecipitation with anti‐Myc antibody, followed by western blotting analysis. The experiment was repeated in triplicates. (E) NIH‐3T3 cells co‐expressing EGFR and either WT MIG6 or the indicated MIG6 mutants were assayed for anchorage‐independent growth in soft agar with EGF (25 ng·mL^−1^). The bar graph depicts the relative colony numbers of MIG6 mutants normalized vector control cells (*n* = 3, mean + SD). Student's *t*‐test was used to determine statistical significance. *, *P* < 0.05; **, *P* < 0.01; and ***, *P* < 0.001.

To investigate the clinical relevance and potential role of MIG6 in tumor progression, we cataloged *ERRFI1* mutations in patient samples by analyzing publicly available data sets, including The Cancer Genome Atlas (TCGA) and Catalogue of Somatic Mutations in Cancer (COSMIC). Out of the 273 curated *ERRFI1* mutations, we further selected mutations based on the following criteria: (a) recurrent mutations, (b) nonsense or frameshift mutations resulting in truncation mutants of MIG6 segment 1/2 domains, or (c) mutations in the region encoding MIG6 segment 1/2 domains (Fig. S3A). Next, we excluded *ERRFI1* mutations co‐occurring with genomic alterations in key oncogenes such as *KRAS*, *BRAF*, *EGFR*, or *PIK3CA* and finalized the list of 25 mutations, as depicted in Fig. 2A. Further functional analysis focused on 13 representative mutations affecting key functional domains and we ectopically co‐expressed the corresponding MIG6 mutants with EGFR in 293 T cells (Fig. 2B).

Consistent with previous findings [19], WT MIG6 effectively suppressed the enzymatic activity of co‐expressed EGFR as shown by reduced p‐EGFR and p‐STAT5 levels (Fig. 2B). In contrast, the MIG6 Y394F/Y395F mutant, in which crucial phosphorylation within the MIG6 segment 2 domain is abrogated, failed to inhibit EGFR activity (Fig. 2B). Under the same experimental conditions, truncated MIG6 mutants, characterized by full deletion of segment 1/2 domains (E184* and R286Qfs*7 mutants), or the partial deletion of the segment 2 domain (E384* and S391* mutants) (Fig. 2A), exhibited reduced efficacy in inhibiting EGFR activity compared to WT MIG6 as demonstrated by p‐EGFR and p‐STAT5 levels (Fig. 2B). Similarly, we observed comparable loss‐of‐function effects in MIG6 mutants carrying mutations within the segment 1/2 domains (A353V, H393R, or Y395Del), while no such effects were observed by mutations in other regions (P75L, L164V, D168N, R202C, S307L, and R453H mutants) (Fig. 2A,B). Co‐immunoprecipitation data demonstrated that while WT MIG6 interacts with EGFR, most of the identified ‘loss‐of‐function’ MIG6 mutants do not bind with EGFR (Fig. 2C). This implies that the compromised inhibition of EGFR by these MIG6 mutants could result from their inability to interact with EGFR, likely due to either the absence of segment 1/2 domains or improper phosphorylation of segment 2 at Y394/Y395 in these MIG6 mutants, as previously reported [19, 22]. Furthermore, this hindered interaction of MIG6 also occurs with the EGFR L858R mutant, accompanied by a failed inhibition of EGFR mutant activity, as confirmed through western blotting analysis (Fig. 2D). This result highlights that the loss‐of‐function effect in these MIG6 mutants extends beyond WT EGFR and likely has a broad impact across all ERBB receptors.

Next, we investigated the functional significance of MIG6 as a tumor suppressor in the context of ligand‐induced EGFR‐mediated transformation. We introduced each MIG6 mutant into NIH‐3T3 cells stably expressing ectopic WT EGFR and assessed the EGF‐induced oncogenic potential using a colony formation assay (Fig. 2E). In line with our previous findings [19], the enforced expression of WT MIG6 significantly inhibited EGFR‐dependent colony formation compared to the control, while MIG6 Y394F/Y395F mutant showed a lesser effect (Fig. 2E). In contrast, all MIG6 mutants, which lacked functional segment 1/2 domains either due to nonsense or frameshift mutations or mutations within the *ERRFI1* region coding these domains (designated as ‘MIG6 Seg1/2 mutants’), showed a significantly reduced ability to suppress colony formation compared to WT MIG6, similar to MIG6 Y394F/Y395F mutant (Fig. 2E). The compromised suppression of colony formation in NIH‐3T3 cells expressing these MIG6 mutants is likely a result of their impaired inhibition of EGFR activity, as confirmed by western blotting analysis showing no significant reduction of p‐EGFR and p‐STAT5 levels (Fig. S3B). Notably, these results are consistent with the data from the prior experiments with 293T cells (Fig. 2B).

In light of the well‐established oncogenic role of EGFRvIII and the EGFR C‐terminal domain deletion mutant in glioblastoma (GBM) [2, 31, 37, 38], we sought to assess whether MIG6 possesses the capability to inhibit the enzymatic activity of these EGFR mutants and mitigate their oncogenic potential. As a prior report [19], MIG6 suppressed the enzymatic activity of the co‐expressed L858R mutant, as evidenced by the reduced levels of p‐EGFR and p‐STAT5 in 293T cells (Fig. S4A). Furthermore, a similar inhibitory effect was observed for both the EGFR CTED8 and EGFRvIII mutants [32], which is associated with their ability to interact with MIG6, as depicted in Fig. S4A,B. Moreover, the similar decrease in colony numbers observed in the soft agar assay conducted with NIH‐3T3 cells co‐expressing the EGFR mutant along with MIG6, in comparison to the outcome of WT EGFR, serves as additional evidence of the impact of MIG6 on the oncogenic potential of these EGFR mutants (Fig. S4C,D).

Taken together, MIG6 Seg1/2 mutants are unable to inhibit EGFR activation and consequently, potentially contribute to aberrant EGFR activation‐mediated cellular transformation in specific genetic contexts.

### 3.3 Deficient tumor suppressor function of MIG6 mutants with impaired segment 1/2 domains

**Fig. 3:**
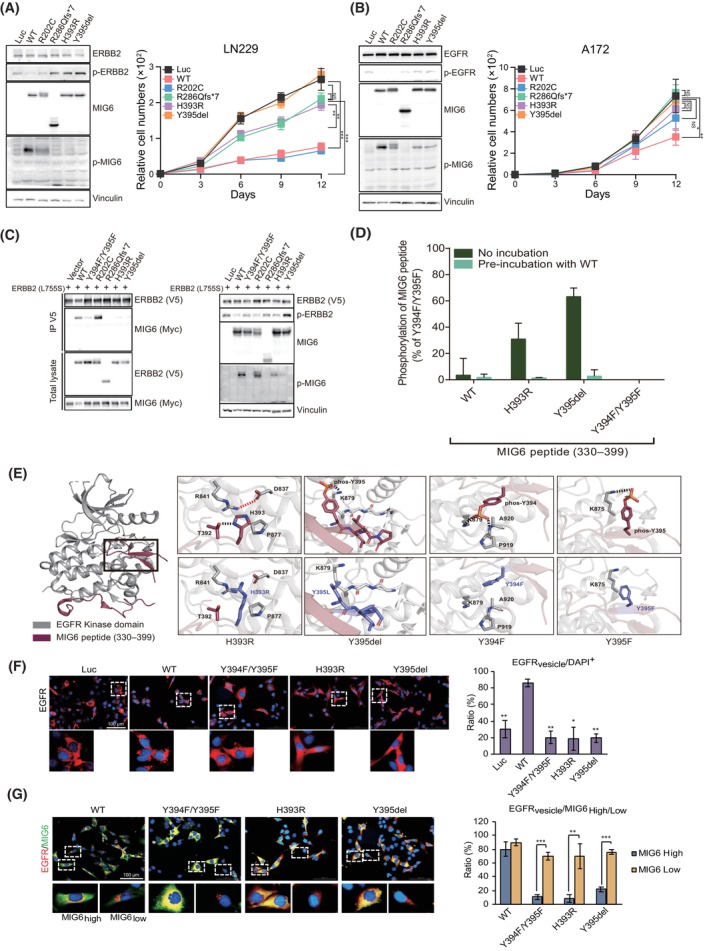
MIG6 mutants with deficient Segment 1/2 domains exhibit loss‐of‐function activity. (A, B) Cell growth of LN229 (A) and A172 (B) cells ectopically expressing either wild‐type (WT) MIG6 or luciferase (Luc), or the indicated MIG6 mutants, was represented with the mean ± SD (*n* = 6). Cell lysates prepared from the same cells were subjected to western blotting analysis with the indicated antibodies. (C) Anti‐V5 immunoprecipitates (left blot) were obtained from the cell lysates prepared from 293T cells co‐expressing V5‐tagged ERBB2 L755S mutant and indicated MIG6 mutants, and the same cell lysates (right blot) were subjected to western blotting analysis with shown antibodies. Each experiment was repeated in triplicates. (D) *In vitro* kinase assay with EGFR kinase and MIG6 peptide (330–399) variants bearing indicated mutations. Relative phosphorylation on the MIG6 peptide was determined by measuring ATP consumption by EGFR unincubated (dark green) and pre‐incubated (light green) with the WT MIG6 peptide (330–399). Three independent experiments with five‐technical replicates were performed and represented in each bar graph. Error bar indicates ± SEM (E) Overall structure (PDB 4ZJV, left) and detailed interactions (upper right) of the complex with EGFR kinase domain (gray) and MIG6 peptide (WT 330–399, brick red), with a particular emphasis on segment 2. Interaction modifications with the EGFR kinase domain were depicted (purple, bottom right) for two patient‐derived mutated residues, H393R and Y395del, as well as for the phosphorylation‐compromised Y394F and Y395F mutated residues. Dashed lines represent ionic interaction (red) hydrogen bonds (black). The structures were displayed using pymol (The PyMOL Molecular Graphic System, Version 3.0 Schrödinger, LCC). (F) Immunocytochemistry with anti‐EGFR in A172 cells expressing indicated MIG6 mutants. Bar graph depicts relative number of cells showing EGFR vesicles normalized to DAPI‐positive cells (*n* = 4, mean ± SD). Statistical significance was assessed relative to WT‐expressing group. Scale bar = 100 um (G) Immunocytochemistry with anti‐EGFR and anti‐MIG6 in A172 cells expressing indicated MIG6 mutants. The number of cells with high‐ or low expression of MIG6 were counted in different four spots and the ratio of cells showing EGFR vesicles were presented in a graph. Bar graph depicts relative number of cells showing EGFR vesicles normalized to DAPI‐positive cells with high‐ or low expression of MIG6 (*n* = 4, mean ± SD). Statistical significance was assessed between MIG6‐highly and lowly expressing groups. Scale bar = 100 μm. EGF (10 ng·mL^−1^) was treated in all panels. In panels A, B, D, F, G, Student's *t*‐test was used to determine statistical significance. *, *P* < 0.05; **, *P* < 0.01; and ***, *P* < 0.001.

To further explore the tumor suppressing role of the characterized loss‐of‐function MIG6 mutants, we selected three representative *ERRFI1* mutations, specifically R286Qfs*7, H393R and Y395del, which result in either MIG6 Seg1/2 mutants or a mutant with impaired phosphorylation at Y394 and Y395 residues. Through retroviral infection, we ectopically introduced the selected three MIG6 mutants or WT MIG6 or R202C mutant in two GBM cell lines (LN229 and A172 cells) where lower expression of MIG6 and EGFR‐dependent cell growth has been confirmed. We assessed the impact of these specific MIG6 Seg1/2 mutants on oncogenic cell proliferation in comparison to the WT MIG6 or R202C mutant (Fig. 3A,B). The inclusion of LN229 cells, carrying the *ErbB2* L755S mutation [39], is particularly important for the functional analysis, enabling examination of MIG6's suppressive effect not only against oncogenic EGFR but also against oncogenic ERBB2 [40].

As expected, a notable reduction of cell growth was observed in both cells overexpressing WT MIG6 and R202C mutant compared to the control (Fig. 3A,B). In contrast, cell lines expressing the MIG6 R286Qfs*7, H393R, or Y395del mutant did not exhibit significant growth inhibition (Fig. 3A,B). The attenuated growth inhibition by MIG6 Seg1/2 mutants may result from their impaired ability to suppress the enzymatic activity of ERBB2 or EGFR in LN229 and A172 cells, respectively, as supported by unaffected p‐EGFR or p‐ERBB2 levels (Fig. 3A,B). To investigate whether the impaired inhibition of ERBB2 L755S mutant and oncogenic cell growth by the MIG6 mutants in LN229 cells is attributed to the lack of interaction between ERBB2 and the introduced MIG6 mutants, we conducted co‐immunoprecipitation in 293T cells co‐transfected with the ERBB2 L755S mutant and the respective MIG6 mutants (Fig. 3C). The results confirm that the MIG6 R286Qfs*7, H393R, and Y395del mutants did not interact with the ERBB2 L755S mutant, resulting in reduced suppression of ERBB2 L755S mutant activity, which was evidenced by the western blotting analysis of p‐ERBB2 and p‐MIG6 levels (Fig. 3C). In contrast, the WT MIG6 and R202C mutant retained their ability to interact with ERBB2 L755S mutant and effectively suppress ERBB2 activity (Fig. 3C). The pivotal role of MIG6 segment 1/2 domains and phosphorylation at Y394/Y395 residues in the tumor suppressing activity of MIG6 was further explored through an *in vitro* kinase assay utilizing purified EGFR L858R mutant and MIG6 GST fusion proteins [19]. The results revealed that EGFR phosphorylation at Y869, which indicates full activation of EGFR, was inhibited when co‐incubated with the GST‐MIG6 segment 1/2, but not with the GST‐MIG6 segment 1/2 with Y394F/Y395F mutations or with GST alone (Fig. S5A,B).

Next, we conducted a more in‐depth analysis of the EGFR inhibitory activity of MIG6 H393R and Y395del mutants using an *in vitro* kinase assay, involving purified EGFR kinase and MIG6 peptides (330–399 amino acids) as substrates. Specifically, we measured ATP consumption in the assay, which reflected the levels of MIG6 peptide phosphorylation at Y394 induced by EGFR kinase. As expected, ATP consumption was not detected in the reaction with MIG6 peptides carrying Y394F/Y395F mutations. In contrast, significantly high levels of ATP consumption occurred in the presence of MIG6 peptides carrying H393R or Y395del mutations compared to WT MIG6 peptides, indicating that the WT MIG6 peptide effectively suppresses EGFR activity, whereas MIG6 peptides carrying H393R or Y395del mutations do not in the *in vitro* kinase assay (Fig. 3D). No additional phosphorylation occurred in any of the peptides after the pre‐incubation with WT MIG6 peptide, indicating complete binding of WT MIG6 to EGFR, physically inhibiting kinase activity of EGFR, and preventing additional phosphorylation on MIG6 peptides.

The impaired inhibitory effect of the MIG6 H393R and Y395del mutants is further elucidated through predicted structural models based on the complexes of the EGFR kinase domain and MIG6 peptide (PDB 4ZJV) (Fig. 3E). In MIG6 segment 2, H393 forms a hydrogen bond with T392 and an aromatic‐π stacking with P877 of EGFR. R841 of EGFR around residue H393 forms an ionic interaction with D837, which is crucial for kinase activity. When H393 is mutated to arginine, the existing interactions are disrupted, and charge repulsion with the positive R841 residue occurs, rendering the structure of MIG6 unable to bind to EGFR and inhibit its function. As reported previously [19], hydrogen bonds are formed between the phosphate group of Y394 in MIG6 with K879 and the backbone of P919 and A920 of EGFR; phosphate group of Y395 in MIG6 with K875 of EGFR. Considering the structural aspects, these two phosphorylation sites on MIG6 segment 2 are crucial for not only enhancing additional interactions with EGFR but also improving its binding stability. Thus, the loss of Y395 phosphorylation due to the MIG6 Y395del mutation could potentially prevent its binding to EGFR and a subsequent reduction in its inhibitory effects on EGFR. Based on these analyses, it is hypothesized that both MIG6 H393R and Y395del mutations may decrease stability between MIG6 segment 2 and EGFR kinase, potentially leading to incomplete inhibition of EGFR enzymatic activity (Fig. 3D).

MIG6 is known to induce the internalization of EGFR, which triggers its migration to late endosomes/lysosomes and promotes its degradation [17, 18, 19, 21, 22]. To explore if the loss‐of‐function MIG6 mutants impact ligand‐stimulated EGFR trafficking, we conducted immunocytochemistry experiments in A172 cells expressing the respective MIG6 mutants (Fig. 3F,G). In line with the previous studies, overexpression of WT MIG6 led to a significant increase in cells (86%) with intracellular EGFR vesicles upon EGF treatment, while cells expressing MIG6 mutants (Y394F/Y395F, H393R, and Y395del) showed diffused cytoplasmic EGFR, indicating impaired endocytosis (Fig. 3F). To dissect the relationship between MIG6 expression and EGFR internalization in the heterogenous context, we compared EGFR vesicle ratios across cells with varying MIG6 levels (Fig. 3G). Interestingly, MIG6 mutants hindered EGF‐induced EGFR internalization only in cells with high MIG6 expression, while having minimal effect in cells with low MIG6 level (Fig. 3G). These findings demonstrate that MIG6 H393R and Y395del mutants exhibit impaired activity in both negative regulatory mechanisms of EGFR by MIG6, which encompass direct enzymatic inhibition of EGFR and the promotion of EGFR degradation. The phenocopy observed in the MIG6 Y394F/Y395F mutant, as well as the H393R and Y395del mutants, further supports the notion that phosphorylation at Y394/Y395 residues is crucial for MIG6's negative feedback function in regulating EGFR at multiple levels (Fig. 3D–G).

### 3.4 Clinical relevance of MIG6 mutant‐mediated tumorigenesis in GBM organoid model

**Fig. 4:**
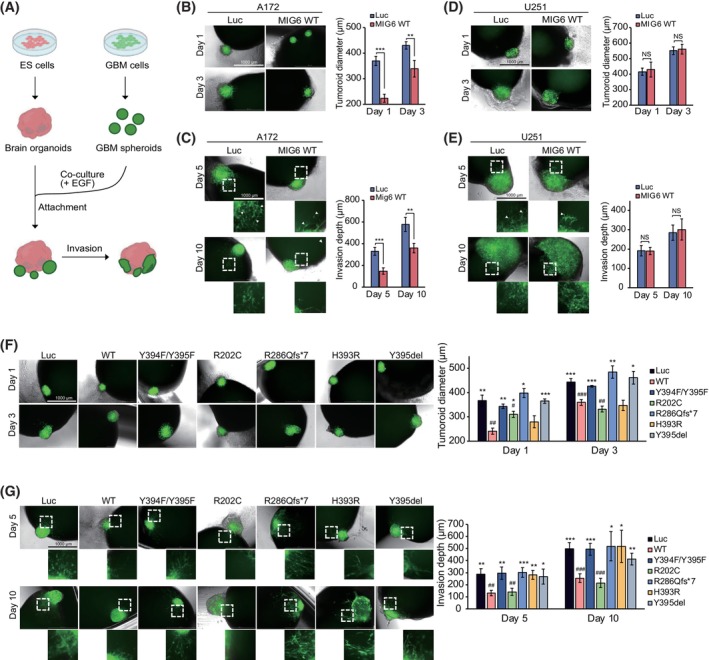
Functional characterization of MIG6‐mediated glioblastoma development in the brain organoid model. (A) Schematic diagram illustrating the generation of the glioblastoma‐brain organoid model. (B–E) Immunofluorescence and brightfield images of brain organoids transplanted with A172 cells (B, C) or U251 cells (D, E) ectopically expressing either wild‐type (WT) MIG6 or luciferase (Luc) were taken at indicated days. Tumor diameters (B and D) and invasion depth into brain organoids (C and E) by transplanted A172 cells or U251 cells were measured and depicted in graphs as mean ± SD. *n* = 3 (B and D) and *n* = 4 (C and E). Statistical significance was assessed between luciferase‐ and WT MIG6‐expressing groups. (F, G) Additional organoid models were generated using the same experimental procedure as described in B–E, but with the inclusion of more A172 cell lines ectopically expressing indicated MIG6 mutants. Subsequently, these established models were characterized, and the tumor diameters (F) and invasion depth into brain organoids (G) by each A172 cell line with different MIG6 genetic background were measured and presented in graphs as mean ± SD. *n* = 3 (F) and *n* = 4 (G). In panel B–G, scale bar = 1 mm. In the graphs, # indicates the statistical significance relative to Luc, and * indicates the statistical significance relative to WT. EGF (10 ng·mL^−1^) was treated in all organoid models described. Student's *t*‐test was used to determine statistical significance. * and #, *P* < 0.05; ** and ##, *P* < 0.01; and *** and ###, *P* < 0.001.

Organoid models have been developed to recapitulate the distinct characteristics of GBM [41]. To gain further insight into the impact of MIG6 activity on GBM development and progression, we employed a physiologically relevant modified cerebral organoid glioma (GLICO) model. In this model, human brain organoids serve as platforms for the transplantation of tumor spheroids, facilitating the establishment of networks between brain organoids and invasive tumor cells [42]. Initially, brain organoids were generated from human embryonic stem cells using methodologies pioneered by the Sasai group [43]. Subsequently, engineered GBM cells overexpressing WT MIG6, or luciferase as a control, were formed into spheroids and co‐cultured with human brain organoids. In this co‐culture system, the GBM spheroids attached to and invaded the brain organoids (Fig. 4A). We found that the overexpression of WT MIG6 led to a reduction in the size of A172 tumor spheroids (Fig. 4B and Fig. S5A) and their integration depth into brain organoids (Fig. 4C). In contrast, EGFR‐negative U251 cells exhibited no discernible phenotypic changes upon MIG6 overexpression (Fig. 4D,E and Fig. S6A). Abrogation of MIG6 expression in LN18 cells by shRNA, which naturally express high levels of MIG6, significantly increased the diameter of LN18 spheroids and exhibited poor integration into brain organoids (Fig. S6B). These findings provide additional support for MIG6's role as a tumor suppressor and emphasize its pivotal function in GBM pathogenesis, particularly in an EGFR‐dependent manner.

Next, we examined the functional consequences of loss‐of‐function MIG6 mutants using a transplanted brain organoid models generated with A172 cells ectopically expressing R286Qfs*7, H393R, Y395del MIG6 mutants, in addition to WT MIG6, MIG6 Y394F/Y395F or R202C mutants employed as control conditions (Fig. 4F,G). Like previous findings, MIG6 R202C mutants exhibited a similar phenotypic effect as WT MIG6, showing a significant reduction in both tumor spheroid size and invasion depth in A172 cells (Fig. 4F,G and Fig. S6C). However, the MIG6 R286Qfs*7, Y395del as well as Y394F/Y395F mutants reversed the inhibitory effects of MIG6 on tumor spheroid size and invasion depth (Fig. 4F,G and Fig. S6C). Meanwhile, the H393R mutant showed a loss‐of‐function only in tumor spheroid invasion, with limited effect on spheroid growth (Fig. 4F,G).

Taken together, these results highlight the critical role of MIG6 segment 1/2 domains and Y394/Y395 phosphorylation in its tumor suppressive function with the brain organoid models. The findings further suggest that *ERRFI1* mutations leading to the deletion of segment 1/2 domains or impaired Y394/Y395 phosphorylation may serve as a key genomic alteration associated with GBM development, especially in the context of specific genetic backgrounds such as EGFR amplification (Note that raw images of immunoblotting data are displayed in Figs S7–S11).

## 4 Discussion

EGFR‐targeted therapy is highly effective for multiple cancer types, including non‐small cell lung cancer, head and neck cancer, and colorectal cancer, particularly when they have *EGFR* genomic changes such as specific somatic mutations, gene amplification, or EGFR protein overexpression [5, 8, 10, 11, 13]. However, current studies suggest that the complexity of EGFR‐driven tumorigenesis extends beyond these genomic alterations [44, 45]. Multiple factors, such as the complex cellular context involving concurrent genomic changes and the interplay of positive and negative regulators of EGFR signaling, are proposed to contribute to the broader perspective of EGFR‐dependent oncogenesis [46, 47, 48, 49, 50]. These factors may also influence the effectiveness of drug responses and potentially contribute to drug resistance against EGFR‐targeted treatment [46, 47, 48].

We previously identified focal *ERRFI1* gene deletions in approximately 20% of GBM cases [19]. Notably, these deletions were predominantly hemizygous and strongly correlated with coexisting *EGFR* amplification [19]. Taking into account these observations and the uncertainty regarding whether *EGFR* amplification and protein overexpression, which are common in GBM, are individually sufficient as definitive causes of cancer development, we hypothesized that aberrant expression or inactivation of MIG6—an EGFR‐negative feedback inhibitor encoded by *ERRFI1*—may potentially serve as an additional genomic alteration leading to tumorigenesis, particularly within the genetic conditions characterized by such EGFR alterations. To test this hypothesis, we systematically investigated the functional consequences of loss and gain of MIG6 activity in the context of EGFR‐mediated oncogenic potential, utilizing engineered GBM cell lines and brain organoids as experimental model systems. Our findings highlight that MIG6 expression levels are crucial in determining oncogenic cell growth, especially in EGFR‐expressing cells. This supports our hypothesis that compromised negative regulation of EGFR by MIG6 due to imbalanced EGFR and MIG6 expression levels could act as a crucial mechanism in EGFR‐driven oncogenesis, even without oncogenic *EGFR* mutations. Although our initial cell line observations suggest potential synergy between increased MIG6 expression and EGFR‐targeted drugs, the relationship between MIG6 expression levels and the drug efficacy remains controversial, requiring further investigation [51, 52, 53].

To further explore the physiological relevance of our finding, we utilized a human brain organoid model transplanted with GBM cells, a modified GLICO model among various GBM organoid models [41]. While the neoplastic cerebral organoid (neoCOR) model is generated by introducing oncogenic mutations into cerebral organoids and is useful for recapitulating the tumor‐initiating stage, the GLICO model exploits the characteristics of GBM‐derived glioma stem cells (GSCs), which are known for their invasive nature [41, 42, 54]. We found that both initial GBM spheroid growth and cell invasion into brain organoids were similar to cell‐based studies. Interestingly, LN18 cells showing the highest MIG6/EGFR ratio failed to integrate into brain organoids despite LN18 spheroids attaching their surface, whereas A172 with a lower MIG6/EGFR ratio spontaneously infiltrated into the inner region of brain organoids. This observation suggests that the invasive potential of GBM cells in the human brain may be influenced by EGFR activity predicted by the MIG6/EGFR ratio. While the organoid‐based model has demonstrated compelling results in establishing the significant role of MIG6 as a key regulator of tumorigenesis, further comprehensive assess of its clinical relevance should be investigated using patient‐derived cells and *in vivo* mouse models in future studies.

The interaction between MIG6 and EGFR, mediated by MIG6 segment 1 domain and phosphorylated segment 2 at Y394/395 residues, has been extensively characterized as a crucial mechanism for MIG6's negative regulation of EGFR [19]. Consistent with the significance of MIG6 segment 1/2 domains, patient‐derived *ERRFI1* mutations involving the deletion of these domains or occurring within the segment 2 domain result in the loss of MIG6's function, resulting in EGFR‐dependent oncogenesis, as validated through both cell and organoid‐based models. Moreover, these MIG6 mutants exhibit compromised activity in promoting EGFR internalization, a key aspect of EGFR's negative regulation by MIG6. This highlights the broad impact of these specific MIG6 mutants on a wide spectrum of outcomes linked to EGFR‐negative regulation.

Upon further characterization of representative *ERRFI1* mutations, specifically focusing on those within the coding region of MIG6 segment 2, we observed slight functional variations in MIG6 mutants based on their locations. For example, the *in vitro* kinase assay demonstrated that both the MIG6 Y395del and H393R mutants exhibited compromised EGFR inhibitory activity, similar to the MIG6 Y394F/Y395F mutant [19]. However, the MIG6 H393R mutant showed fewer effects compared to the Y395del mutant in various aspects, including enzymatic inhibition of EGFR, cell proliferation, and tumor spheroid growth in organoid models. Based on structural modeling‐based predictions, it is suggested that while both H393R and Y395del mutations in segment 2 domain weakens interaction stability with the EGFR kinase domain, the Y395del mutation has a more significant effect due to the absence of essential Y395 phosphorylation necessary for these interactions [19].

## 5 Conclusions

In conclusion, we provide compelling data indicating that an imbalanced stochiometric ratio between MIG6 and EGFR may represent a crucial mechanism associated with dysregulated EGFR‐dependent tumorigenesis. Furthermore, we have discovered that a subset of *ERRFI1* mutations derived from patients, specifically those deleting MIG6 segment 1 and 2 domains or occurring within the region encoding the segment 2 domain, exhibited characteristics indicative of loss‐of‐function mutations, consequently contributing to aberrant EGFR activation. Thus, both the status of MIG6 expression and *ERFFI1* mutations could serve as additional biomarkers, alongside well‐established *EGFR* mutation status, to select patients who may benefit from EGFR‐targeted cancer therapy, particularly in cases where known oncogenic *EGFR* mutations are absent.

## Conflict of interest

The authors declare no conflict of interest.

## Author contributions

Designed research: SAY, J‐WH, H‐SC, and JC. Performed research: DC, SAY, SK, HK, MKC, HC, and AL. Contributed new reagents/analytic tools: SS, SY, JKJ, and DEY. Analyzed data: SAY, J‐WH, HSC, KK, and JC. Wrote the paper: SAY, H‐SC, HJC, and JC. All authors have reviewed and approved the final manuscript.

### Peer review

The peer review history for this article is available at https://www.webofscience.com/api/gateway/wos/peer‐review/10.1002/1878‐0261.13717.

## Supporting information


**Fig. S1.** The stoichiometric ratio of MIG6 and EGFR expression correlates with EGFR‐dependent oncogenic growth in a subset of GBM and lung cancer cell lines.
**Fig. S2.** Effects of EGFR/ERBB2 targeted drugs on the growth of EGFR‐positive and negative glioblastoma cells.
**Fig. S3.** Selection and characterization of patient‐derived ERRFI1 mutations.
**Fig. S4.** Characterization of inhibitory effects of oncogenic activity of EGFR mutants by MIG6.
**Fig. S5.** MIG6 segment 1/2 domains and Y394/395 phosphorylation are crucial for EGFR enzymatic inhibition.
**Fig. S6.** Phenotypic effects of WT MIG6 and loss of function MIG6 mutants on the glioblastoma‐brain organoid model.
**Fig. S7.** Raw images for western blotting data of Fig. 2.
**Fig. S8.** Raw images for western blotting data of Fig. 3.
**Fig. S9.** Raw images for western blotting data of Fig. S3B.
**Fig. S10.** Raw images for western blotting data of Fig. S4.
**Fig. S11.** Raw images for western blotting data of Fig. S5B.
**Table S1.** Composition of buffers used in this study.

## Data Availability

The data that support the findings of this study are available in Figs S1–S6. Raw images of immunoblotting data are displayed in Figs S7–S11.
