# Supplementary material for: Functional loss of ERBB receptor feedback inhibitor 1 (MIG6) promotes glioblastoma tumorigenesis by aberrant activation of epidermal growth factor receptor (EGFR)
Source: Mol Oncol. 2024 Aug 11;19(3):937–53. doi: 10.1002/1878-0261.13717 (PMC11887669; doi:10.1002/1878-0261.13717)
Supplement: Supplementary file 1 — Fig. S1. The stoichiometric ratio of MIG6 and EGFR expression correlates with EGFR‐dependent oncogenic growth in a subset of GBM and lung cancer cell lines. Fig. S2. Effects of EGFR/ERBB2 targeted drugs on the growth of EGFR‐positive and negative glioblastoma cells. Fig. S3. Selection and characterization of patient‐derived ERRFI1 mutations. Fig. S4. Characterization of inhibitory effects of oncogenic activity of EGFR mutants by MIG6. Fig. S5. MIG6 segment 1/2 domains and Y394/395 phosphorylation are crucial for EGFR enzymatic inhibition. Fig. S6. Phenotypic effects of WT MIG6 and loss of function MIG6 mutants on the glioblastoma‐brain organoid model. Fig. S7. Raw images for western blotting data of Fig. 2. Fig. S8. Raw images for western blotting data of Fig. 3. Fig. S9. Raw images for western blotting data of Fig. S3B. Fig. S10. Raw images for western blotting data of Fig. S4. Fig. S11. Raw images for western blotting data of Fig. S5B. Table S1. Composition of buffers used in this study. [file MOL2-19-937-s001.docx]

**Supplementary Table S1. Composition of buffers used in this study**

| **Buffer name** | **Experiments** | **Composition** |
| --- | --- | --- |
| RIPA buffer | Immunoblotting, Immunoprecipitation | 50 mM Tris‐HCl, pH 7.4, 150 mM NaCl, 5 mM EDTA, 1% NP‐40, 0.5 mM sodium deoxycholate, 0.1% SDS |
| NP40 buffer | Immunoblotting, Immunoprecipitation | 50 mM Tris-HCl pH 7.4, 1% NP-40, 140 mM NaCl, 5 mM EDTA |
| Lysis buffer | Purification of Proteins and Peptides | 300 mM NaCl, 25 mM Tris pH 7.5, 30 mM Imidazole, 10% Glycerol with 0.5 mM TCEP and protease inhibitor cocktail |
| Elution buffer | Purification of Proteins and Peptides | 300 mM NaCl, 25 mM Tris-HCl pH 7.5, 10% Glycerol, 300 mM imidazole with 0.5 mM TCEP |
| SEC buffer | Purification of Proteins and Peptides | 20 mM Tris-HCl pH 7.5, 300 mM NaCl, 0.5mM TCEP |
| Kinase reaction buffer | *In vitro* Kinase Assay | 40 mM Tris-HCl pH7.5, 20 mM MgCl_2_, 0.1 mg/ml BSA, 2 mM MnCl_2,_ and 1 mM TCEP |

**Supplementary Figures**


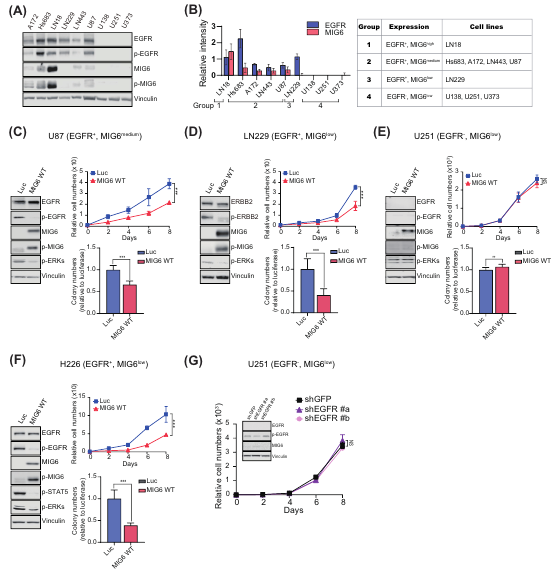


**Fig. S1. The stoichiometric ratio of MIG6 and EGFR expression correlates with EGFR-dependent oncogenic growth in a subset of GBM and lung cancer cell lines.**

(A) Expression levels of the indicated proteins were assessed through immunoblotting with lysates from nine glioblastoma cell lines treated with 10 ng/ml EGF for 15 minutes, using specific antibodies. (B) A graph depicting the comparative protein levels of EGFR and MIG6 across 9 GBM cell lines. Protein quantities are presented as the mean ±SEM (n=2), calculated from Western blotting bands of EGFR or MIG6 that were standardized to vinculin using LI-COR Image Studio. A table indicates the categorization of cell lines into four groups based on their EGFR and MIG6 expression levels. (C-F) Graphs show relative cell numbers of U87 (C), LN229 (D), U251 (E), and H226 (F) cells expressing WT MIG6 (red) or luciferase (Luc) (blue) over time, normalized by the number of cells at day 0, represented as the mean ±SD of octuplicate (top right panel). The relative colony numbers formed in soft agar by the cells expressing WT MIG6 (red), quantified following 2 weeks and normalized with the colony numbers of the cells expressing Luc (blue) were depicted as the mean ± SD of triplicates (bottom right panel). Immunoblotting images were obtained through the analysis of cell lysates prepared from the aforementioned cells, utilizing the indicated antibodies. The phosphorylated residues corresponding to the indicated proteins are detected using the following phospho-specific antibodies: p-EGFR (Y1092), p-MIG6 (Y394/Y395), p-STAT5 (Y694), and p-ERKs (T202/Y204). (G) Relative cell numbers of U251 cells expressing either shRNAs targeting EGFR (purple and pink) or GFP (black) are shown at the indicated days, normalized to the cell numbers at day 0. Western blotting images were acquired from lysates of each cell using the specified antibodies. EGF was treated in all cells described in Figure 1 before preparing cell lysates (10 ng/mL for 15 minutes).


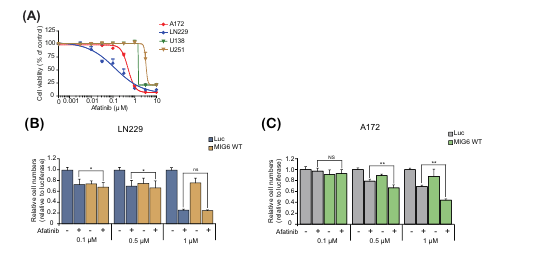


**Fig. S2. Effects of EGFR/ERBB2 targeted drugs on the growth of EGFR-positive and negative glioblastoma cells.**

(A) A172, LN229, U138, and U251 cells were treated with afatinib at the indicated concentrations for 7 days in the absence of EGF, and their cell viability was evaluated. The results are presented in a graph as the mean ±SD of octuplicate wells and are representative of three independent experiments. (B and C) Relative cell numbers of LN229 (B) and A172 (C) cells ectopically expressing luciferase (Luc) or WT MIG6 was evaluated following the treatment of the indicated concentrations of dacomitinib or afatinib for 4 days. EGF was treated as 10 ng/mL. The graphs were normalized using luciferase-expressing cells under each drug treatment condition.


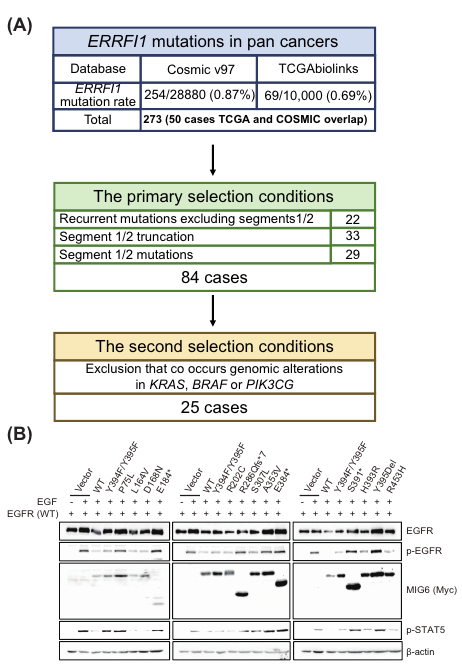


**Fig. S3. Selection and characterization of patient-derived EFFRI mutations.**

(A) Schematic illustrating the criteria used for selecting ERFFI1 mutations for functional characterization from publicly available cancer genome databases (COSMIC and TCGA). (B) Cell lysates prepared from NIH-3T3 cells co-expressing WT EGFR and either MYC-tagged WT MIG6 or indicated MIG6 mutants, following EGF treatment, were blotted with antibodies against EGFR, p-EGFR (Y1092), or p-STAT5 (Y694). β-actin was used as a loading control.


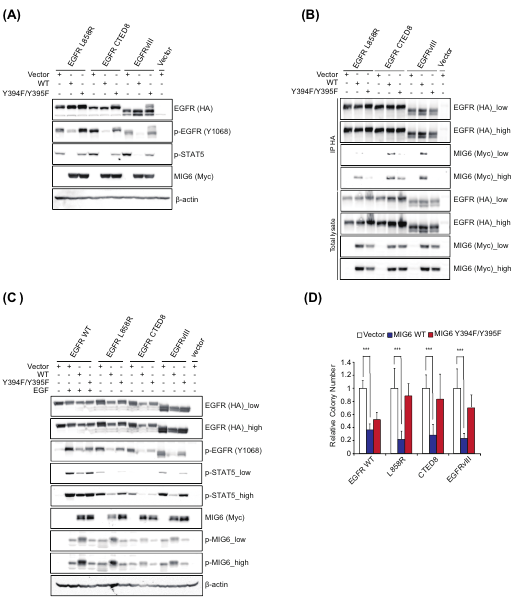


**Fig. S4. Characterization of inhibitory effects of oncogenic activity of EGFR mutants by MIG6.**

(A) Cell lysates from 293T cells co-expressing HA-tagged EGFR L858R, CTED8, EGFRvIII mutants, and either Myc-tagged WT MIG6 or Y394F/Y395F mutant were prepared. Following EGF treatment, the lysates were subjected to immunoblotting using antibodies against HA, Myc, p-EGFR, or p-STAT5 with β-actin utilized as a loading control. **(B)** Immunoprecipitation was performed with an anti-HA antibody using cell lysates described in (A), followed by immunoblotting with the indicated antibodies. (C) ​Cell lysates were prepared from NIH-3T3 cells co-expressing WT EGFR, EGFR L858R, EGFR CTED8, EGFRvIII mutants, and either MYC-tagged WT MIG6 or Y394F/Y395F mutants after EGF treatment. Subsequently, the lysates underwent immunoblotting using the identical antibodies as described in (A). (D) NIH-3T3 cells described in (C) were utilized for anchorage-independent growth in soft agar with EGF (25 ng/mL). The bar graph illustrates the relative colony numbers of MIG6 mutants normalized vector control cells (*n* = 3, mean + SD).


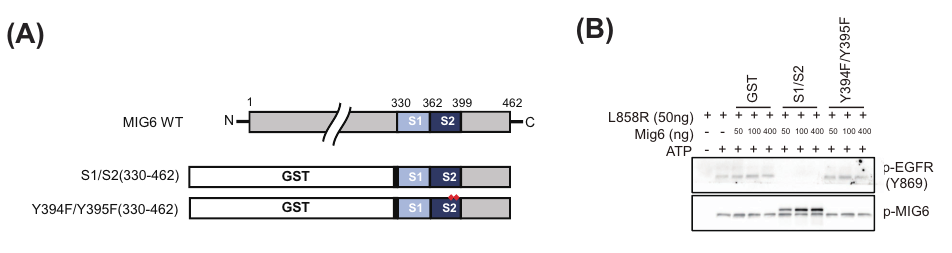


**Fig. S5. MIG6 segment 1/2 domains and Y394/395 phosphorylation are crucial for EGFR enzymatic inhibition.**

(A) Diagram illustrating GST-fused MIG6 proteins, purified for in vitro kinase assays, encompassing segment 1 and 2 domains (S1/S2) or variants with mutations on the two tyrosine residues in segment 2 (Y394F/Y395F). (B) Phosphorylation levels of EGFR at Y869 and MIG6 at Y394/Y395 were examined by Western blotting with phospho-specific antibodies, followed by an in vitro kinase assay involving purified EGFR (L858R) and GST-MIG6 fusion proteins, as depicted in Figure A.


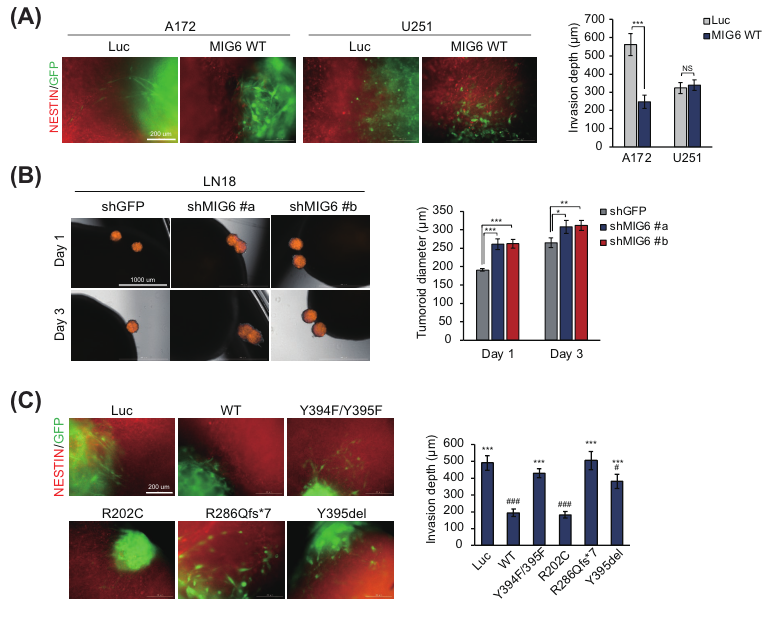


**Fig. S6. Phenotypic effects of WT MIG6 and loss of function MIG6 mutants on the glioblastoma-brain organoid model.**

(A) Immunofluorescence of brain organoids transplanted with A172 or U251 cells overexpressing wild-type MIG6. A172 and U251 cells were labelled with GFP and immunostained NESTIN was used as a neural marker or brain organoid. Graph indicates the invasion depth measured by the length of GFP signal. (B) Immunofluorescence and brightfield of brain organoids transplanted with LN18 cells expressing shRNA targeting MIG6. Graph indicates the diameters of LN18 spheroids. (C) Immunofluorescence of brain organoids transplanted with A172 cells overexpressing indicated MIG6 mutants. Graph indicates the invasion depth measured by the length of GFP signal. In C, # indicates the statistical significance relative to Luc and * indicates the statistical significance relative to WT. EGF (10 ng/mL) was treated in all panels.

**
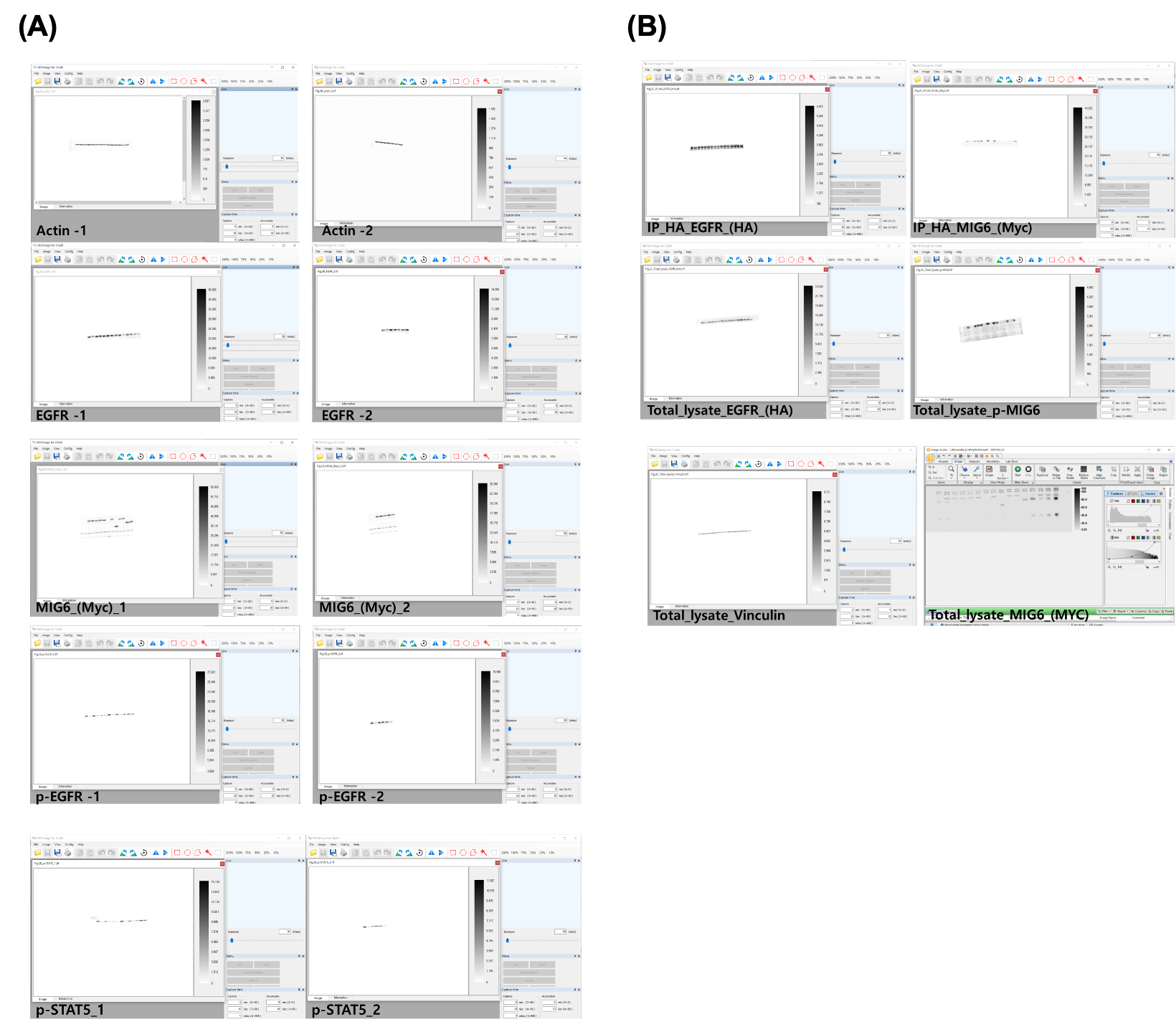
**

**Fig. S7. Raw images for western blotting data of Fig. 2.**

(A) Raw data images from developer for Fig. 2B. (B) Raw data images from developer for Fig. 2C.


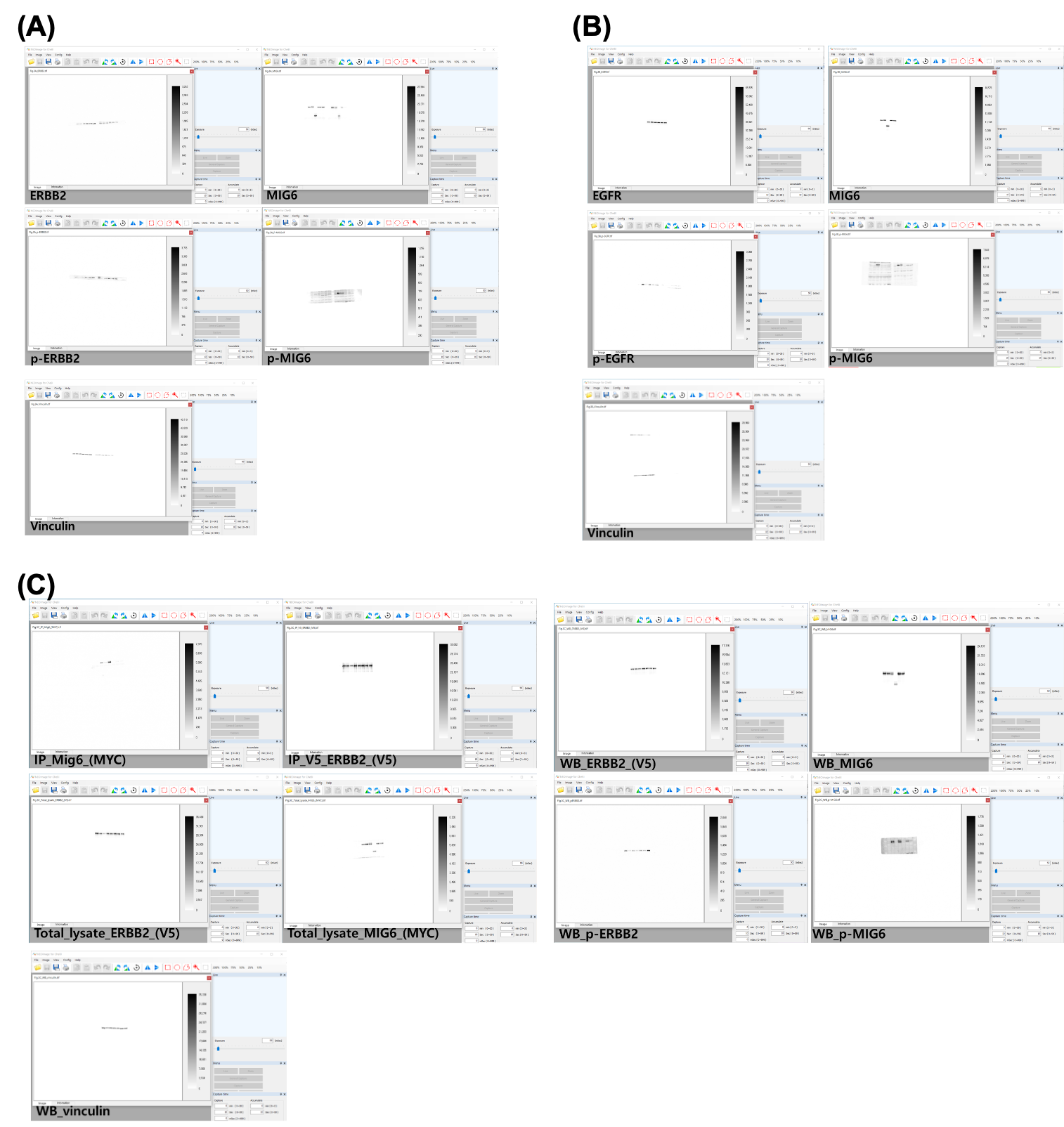


**Fig. S8. Raw images for western blotting data of Fig. 3.**

(A) Raw data images from developer for Fig. 3A. (B) Raw data images from developer for Fig. 3B. (C) Raw data images from developer for Fig. 3C.


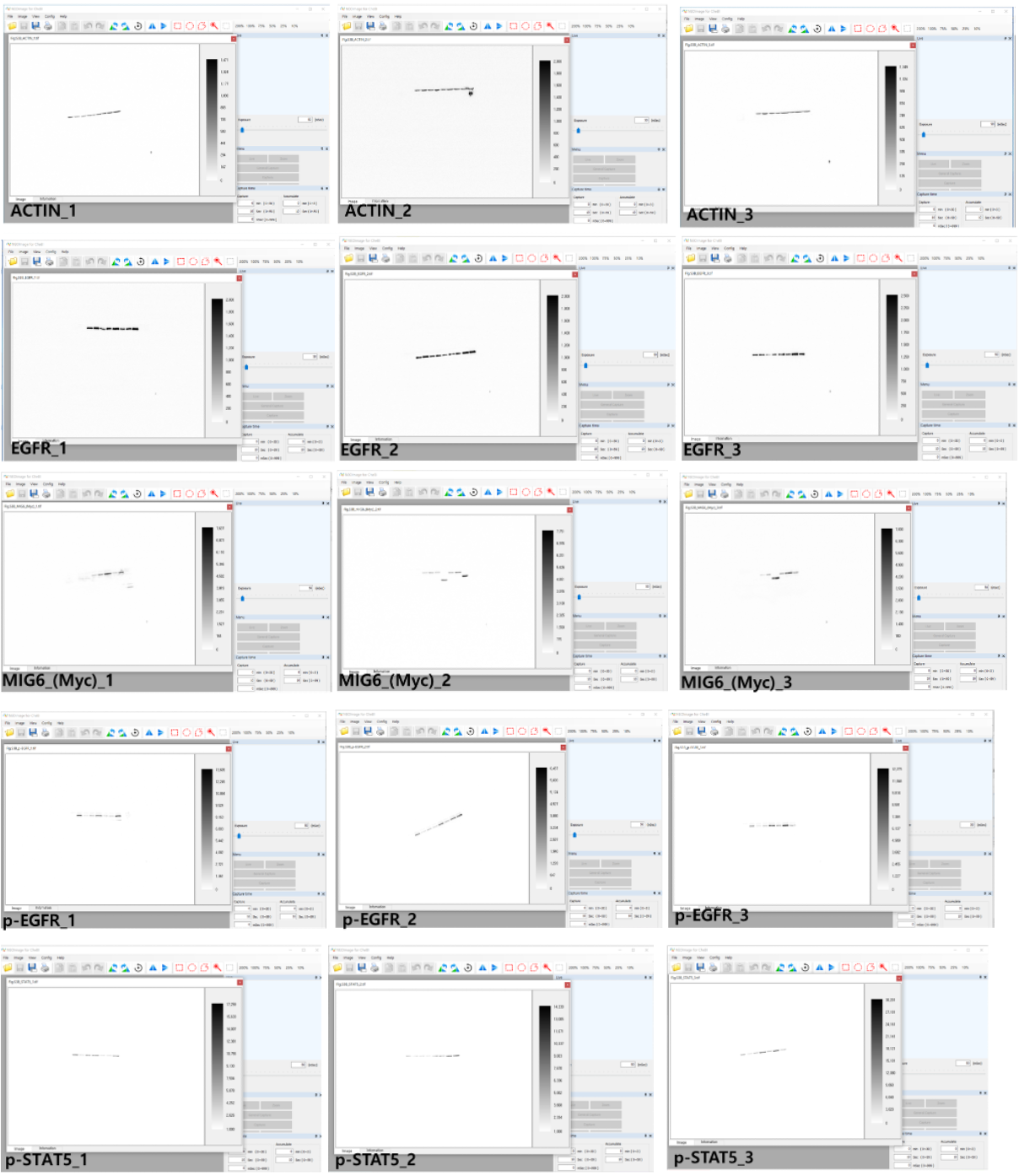


**Fig. S9. Raw images for western blotting data of Fig. S3B.**


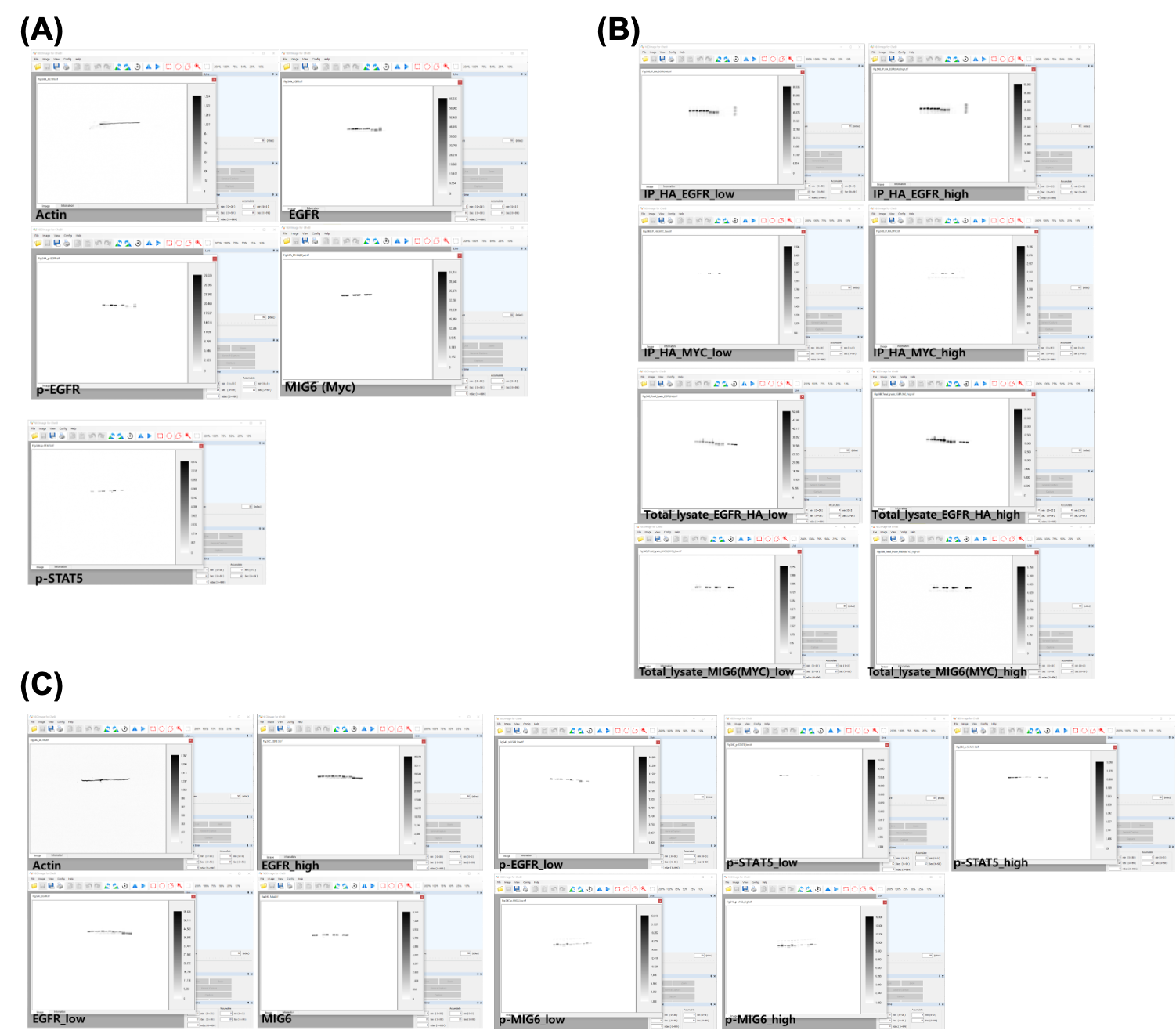


**Fig. S10. Raw images for western blotting data of Fig. S4.**

(A) Raw data images from developer for Fig. S4A. (B) Raw data images from developer for Fig. S4B. (C) Raw data images from developer for Fig. S4C.


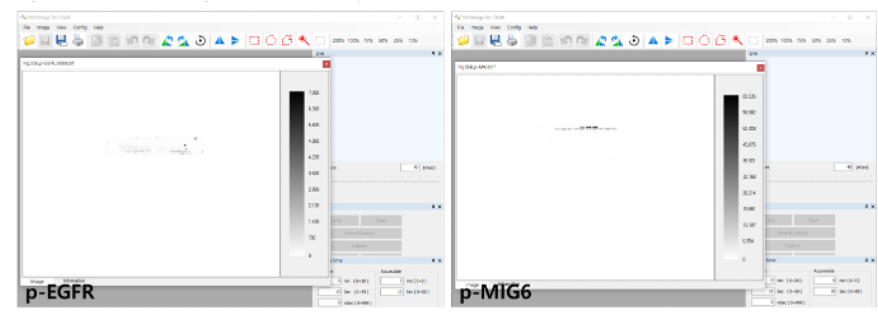


**Fig. S11. Raw images for western blotting data of Fig. S5B.**
